# Alcohol consumption and the risk of postoperative mortality and morbidity after primary hip or knee arthroplasty – A register-based cohort study

**DOI:** 10.1371/journal.pone.0173083

**Published:** 2017-03-17

**Authors:** Torill A. Rotevatn, Henrik Bøggild, Christinna R. Olesen, Christian Torp-Pedersen, Rikke N. Mortensen, Per F. Jensen, Charlotte Overgaard

**Affiliations:** 1 Department of Health Science and Technology, Aalborg University, Aalborg, Denmark; 2 Department of Clinical Epidemiology, Aalborg University Hospital, Aalborg, Denmark; 3 Department of Anesthesia, Næstved Hospital, Næstved, Denmark; Providence VA Medical Center, UNITED STATES

## Abstract

**Objective:**

To investigate the implications of low and moderate preoperative alcohol consumption on postoperative mortality and morbidity after primary hip and knee arthroplasty.

**Methods:**

A total of 30,799 patients who underwent primary hip or knee arthroplasty between January 1^st^, 2005 and October 8^th^, 2011 with information on preoperative alcohol consumption (0 grams of pure alcohol/week, >0–168 g/week, >168–252 g/week, and >252 g/week) were identified through the Danish Anesthesia Database. The 90-day and 1-year risks of mortality (primary outcomes), 1-year risk of prosthetic infection, and 30-day risks of cardiovascular disease and deep venous thrombosis (secondary outcomes) were estimated by Cox regression analysis.

**Results:**

We identified 285 (0.9%) deaths within the first 90 days and 694 (2.3%) within the first year. Within the first 30 days, 209 (0.7%) and 270 (0.9%) patients had acquired cardiovascular disease and deep venous thrombosis, respectively, and 514 (1.7%) patients developed prosthetic infection within the first year. The adjusted mortality models yielded hazard ratios of 0.55 (95% confidence interval [CI] 0.41 to 0.74) at 90 days and 0.61 (95% CI 0.51 to 0.73) at 1 year for the group consuming >0–168 g/week when compared to abstainers. Adjusted hazard ratios showed that the group consuming >0–168 g/week had a 0.91 (95% CI 0.75 to 1.11) risk of prosthetic infection, 0.68 (95% CI 0.50 to 0.92) risk of cardiovascular disease and 0.88 (95% CI 0.67 to 1.15) risk of deep venous thrombosis when compared to abstainers.

**Conclusions:**

This study demonstrates that low-to-moderate alcohol consumption prior to primary hip or knee arthroplasty is associated with lower risks of mortality at both 90 days and 1 year after surgery and of cardiovascular disease after 30 days. More research from longitudinal studies is needed to identify specific causal relations and explanations.

## Introduction

Excessive use of alcohol prior to surgery is an established risk factor for postoperative mortality and morbidity [1]. Most patients referred for elective surgery have a low or moderate use of alcohol [2], but the importance of this level of consumption on adverse postoperative outcomes is uncertain [3]. In many studies, abstainers are grouped together with low and moderate alcohol consumers, and several studies have investigated a combination of multiple surgery populations with uneven risk profiles [3]. A recent meta-analysis by Eliasen et al. (2013) concluded that preoperative alcohol consumption was associated with an increased risk of postoperative morbidity but not with postoperative mortality [3]. Furthermore, low-to-moderate consumption did not seem to be associated with postoperative complications, but the few existing studies that included this consumption group reported lower mortality risks when this group was compared to abstainers [3]. However, in addition to the general tendency of grouping abstainers together with low-to-moderate consumers, Eliasen et al. (2013) pinpoints that several studies tend to lack sufficient confounder adjustment [3]. This gap in knowledge regarding postoperative mortality and morbidity related to a low-to-moderate alcohol consumption may lead to unjustified advice to patients. More knowledge on the subject is essential to improve preoperative risk management.

To address the importance of preoperative alcohol consumption in the prognosis of adverse postoperative outcomes without excessive confounding by multiple risk profiles, we selected an orthopedic population that underwent elective primary hip or knee arthroplasty that have received little attention in previous research [3,4]. Therefore, the aim of this study was to investigate the relationship between preoperative alcohol consumption, with a focus on abstainers and low-to-moderate consumers, and the risks of postoperative mortality and morbidity following primary hip or knee arthroplasty. Based on the existing knowledge, we expected a decreased risk of mortality among low-to-moderate alcohol consumers when compared to abstainers and a lack of risk difference between these groups regarding postoperative morbidity.

## Methods

### Data sources

The Danish Anesthesia Database in conjunction with several other nationwide registers was used to conduct this large, register-based cohort study [5,6]. The Danish Anesthesia Database is a clinical quality database that prospectively and consecutively collects anesthetic information on patients’ perioperative periods from Danish departments of anesthesia [7]. The database covered fourteen departments in 2005 and was later increased to a coverage ratio of 70% and 40 departments [7,8]. The Danish Anesthesia Database allowed the identification of date and type of surgery (Nordic Medico Statistical Committee (NOMESCO) classification of surgical procedures) [9], weekly alcohol consumption, smoking status, American Society of Anesthesiologists physical status classification [10], height and weight. Health professionals collect the information through a uniform questionnaire whose completion is required prior to surgical procedures in Denmark. The National Patient Register allowed the identification of birth year and sex in addition to pre- and postoperative admission diagnoses (International Classification of Diseases (ICD) 8^th^ and 10^th^ revision) from all hospitals in Denmark [5,6,11]. Survival status was identified through The Register of Causes of Death, in which all deaths in Denmark are registered [12]. Data on preoperative medication use were identified though The National Prescription Registry, where all claimed prescriptions from Danish pharmacies are registered with World Health Organization (WHO)’s Anatomical Therapeutic Chemical (ATC) classification codes [13,14]. Data on annual household income were accessible via The Danish Income Statistics Register [6]. Data were accessed through Statistics Denmark and were combined across registries through individual linkage using Danish Civil Personal Register numbers [5,15].

### Study population

We included all patients who were reported as having undergone primary hip or knee arthroplasty (NOMESCO: *KNFB* or *KNGB*) in Denmark from January 1^th^, 2005 to October 8^th^, 2011 through the Danish Anesthesia Database. We only included the first entry of either primary hip or knee arthroplasty for each patient. Subsequently, only procedures coded as elective were included due to the very different risk profiles in acute operations [16,17]. Due to general risk differences, only patients who were over 45 years old [18,19] and who had an American Society of Anesthesiologists physical status classification and a Charlson Comorbidity Index of less than 4 were included to improve internal comparability [16,20].

### Covariates

Anesthesiologists were ticking boxes with categorical data on alcohol consumption on a form, and this information is directly coded into the registry.

Information on alcohol consumption was taken directly from the registry (unit) and was transformed into grams of pure alcohol (one unit corresponds to 12 grams of pure alcohol in Denmark): 0 grams of pure alcohol/week (abstention), >0–168 g/week (low-to-moderate consumption), >168–252 g/week (high consumption), or >252 g/week (excessive consumption). In addition to age and sex, other potential confounders were identified, including smoking status [21,22], socioeconomic status [23–25], preoperative comorbidity status [16,20,23], and body mass index (BMI) [26]. Age was divided into quartiles: 45 to 62.4 years, 62.4 to 69.2 years, 69.3 to 76.2 years and over 76.2 years. Smoking status was recorded as smoker, former smoker, non-smoker, or not asked. Many alcohol consumers are smokers [27]. Consequently, we grouped patients not asked about their smoking status as current smokers, as information on smoking status was lacking for a relatively large group of excessive alcohol consumers. Sensitivity analyses were conducted to control the robustness of this decision. Annual household income the year preceding surgery was divided into quartiles and was used as a proxy for socioeconomic status. Preoperative hospital diagnoses defined the Charlson Comorbidity Index [28]. The Charlson Comorbidity Index and American Society of Anesthesiologists physical status classification represented the preoperative comorbidity status. BMI was calculated using information on height and weight and was categorized according to the WHO classification [29]. Patients with BMIs above 60 or below 13 were categorized as missing to exclude possible incorrect entries of height and weight. Hip or knee arthroplasty are frequent in patients with rheumatoid arthritis, and these patients may have received treatment with methotrexate, which requires restriction of alcohol consumption [30]. Therefore, information on preoperative use of methotrexate (ATC code: L01BA01, L04AX03) was included.

### Outcomes

The follow-up periods for all outcomes were set according to the European Perioperative Clinical Outcome (EPCO) definitions [31]. The 90-day and 1-year risks of mortality were set as primary outcomes. Prosthetic infection (ICD-10: T845-847), cardiovascular disease (ICD-10: I63-64, G458-459, I21-22) and deep venous thrombosis (ICD-10: I801-803, I809) were set as secondary outcomes, as these postoperative complications have especially been associated with patients with high or excessive alcohol consumption or alcohol abuse [1,4,32–34]. To comply with the EPCO guidelines, the 1-year risk of prosthetic infection and 30-day risks of cardiovascular disease and deep venous thrombosis were examined. Cases and dates of postoperative readmissions caused by these diagnoses were identified. Participants were followed until the date of event or December 31, 2012.

### Statistical analysis

Differences in baseline characteristics between alcohol consumption levels were tested with Pearson’s chi-squared test. The Kaplan Meier survival function was used to estimate 1-year survival curves and cumulative death rates for different levels of alcohol consumption. The Aalen-Johansen estimator was used to estimate the cumulative incidence of cardiovascular disease and deep venous thrombosis 30 days postoperative and 1 year after surgery for prosthetic infection. Cox Proportional Hazards Regression was used to estimate unadjusted and adjusted hazard ratios (HRs) with corresponding 95% confidence intervals (CIs). All results were based on a complete case analysis. Analyses were adjusted for age, sex, smoking status, BMI, annual income, Charlson Comorbidity Index, American Society of Anesthesiologists physical status classification, operation type and preoperative use of methotrexate. The proportional hazards assumption was validated with Schoenfeldt-residuals [35]. The Charlson Comorbidity Index did not meet this assumption, and adjusted models were stratified according to this variable. Interactions between alcohol consumption and smoking status, alcohol consumption and sex, alcohol consumption and age, and alcohol consumption and comorbidity status were examined. No statistical significant interactions were detected. In subgroups defined by age-quartiles (45 to 62.4 years, 62.5 to 69.2 years, 69.3 to 76.2 years and over 76.2 years) we furthermore conducted Cox regression analyses for 1-year mortality to identify possible risk-differences in age groups. The subgroup analyses were adjusted for sex, smoking status, BMI, annual income, Charlson Comorbidity Index, American Society of Anesthesiologists physical status classification, operation type and preoperative use of methotrexate. Sensitivity analyses were conducted to identify possible changes in the risk estimates when patients not asked about their smoking status were grouped as non-smokers. An additional sensitivity analysis was conducted when abstaining patients not asked about their smoking status were grouped as non-smokers while the remaining patients not asked were grouped as smokers. All significance was set at P<0.05.

The SAS statistical software package for Windows, version 9.4 (SAS Institute, Cary, NC, USA), was used for data management, and the R statistical software package, version 3.0.2 (R Development Core Team), was used for statistical analyses.

### Ethics

Use of data for this study was approved by the Danish Data Protection Agency (No. 2007-41-1667). In Denmark register-based studies do not require ethical approval or obtained written informed consent [36].

## Results

### Participants

There were 48,817 registrations of primary hip or knee arthroplasty in the study period. When only first operations were included, 42,904 patients were eligible for the study. Of these, 5,770 (13.5%) operations were registered as acute, 1,233 (2.9%) patients were under 45 years, 169 (0.4%) had an American Society of Anesthesiologists physical status classification of more than 3 and 170 (0.4%) had a Charlson Comorbidity Index of more than 3, any of which led to their exclusion. Of the 35,562 remaining patients, 4,015 (11.3%) were missing data on alcohol consumption, 407 (1.1%) on BMI, 324 (0.9%) on American Society of Anesthesiologists physical status classification, 10 (0.03%) on annual income, and five (0.01%) on smoking status and were therefore excluded from further analyses. Two (0.01%) patients were additionally excluded due to incorrect entries of dates. These exclusions resulted in a final study population of 30,799 patients.

The final study population consisted of 13,525 (43.9%) abstainers, 12,853 (41.7%) low-to-moderate consumers (>0–168 g/week), 1,958 (6.4%) high consumers (>168–252 g/week), and 2,463 (8.0%) excessive consumers (>252 g/week). The baseline characteristics for the consumption groups are presented in Table 1.

**Table 1:** Baseline characteristics (*n* (%)) among 30,799 patients undergoing primary hip or knee arthroplasty in the period from January 2005 to October 2011, presented according to alcohol consumption levels and in total.

	Abstention^a^(n = 13,525)	Low-to-moderate^a^(n = 12,853)	High^a^(n = 1,958)	Excessive^a^(n = 2,463)	Total(n = 30,799)	P value^b^
**Age**						
45 to 62.4	3,113 (23.0)	3,265 (25.4)	566 (28.9)	756 (30.7)	7,700 (25.0)	
62.5 to 69.2	3,024 (22.4)	3,343 (26.0)	636 (32.5)	695 (28.2)	7,698 (25.0)	
69.3 to 76.2	3,226 (23.9)	3,444 (26.8)	489 (25.0)	543 (22.1)	7,702 (25.0)	
Over 76.2	4,162 (30.8)	2,801 (21.8)	267 (13.6)	469 (19.0)	7,699 (25.0)	<0.0001
**Gender**						
Women	9,665 (71.5)	7,145 (55.6)	475 (24.3)	1,017 (41.3)	18,302 (59.4)	
Men	3,860 (28.5)	5,708 (44.4)	1,483 (75.7)	1,446 (58.7)	12,497 (40.6)	<0.0001
**Surgery type**						
Hip arthroplasty	7,076 (52.3)	7,020 (54.6)	1,085 (55.4)	1,246 (50.6)	16,427 (53.3)	
Knee arthroplasty	6,449 (47.7)	5,833 (45.4)	873 (44.6)	1,217 (49.4)	14,372 (46.7)	<0.0001
**Smoking status**						
Current	2,078 (15.4)	2,365 (18.4)	512 (26.1)	505 (20.5)	5,460 (17.7)	
Former	2,267 (16.8)	3,763 (29.3)	629 (32.1)	461 (18.7)	7,120 (23.1)	
Non-smoker	7,445 (55.0)	6,129 (47.7)	725 (37.0)	510 (20.7)	14,809 (48.1)	
Not asked	1,735 (12.8)	596 (4.6)	92 (4.7)	987 (40.1)	3,410 (11.1)	<0.0001
**BMI**						
Underweight	262 (1.9)	133 (1.0)	19 (1.0)	33 (1.3)	447 (1.5)	
Normal weight	3,995 (29.5)	3,967 (30.9)	534 (27.3)	673 (27.3)	9,169 (29.8)	
Overweight	4,914 (36.3)	5,304 (41.3)	886 (45.3)	998 (40.5)	12,102 (39.3)	
Obese	4,354 (32.2)	3,449 (26.8)	519 (26.5)	759 (30.8)	9,081 (29.5)	<0.0001
**Annual income**^c^						
≤210	4,423 (32.7)	2,458 (19.1)	278 (14.2)	598 (24.3)	7,757 (25.2)	
>210 to 300	3,590 (26.5)	3,084 (24.0)	346 (17.7)	501 (20.3)	7,521 (24.4)	
>300 to 480	3,017 (22.3)	3,499 (27.2)	561 (28.7)	658 (26.7)	7,735 (25.1)	
>480	2,495 (18.4)	3,812 (29.7)	773 (39.5)	706 (28.7)	7,786 (25.3)	<0.0001
**CCI**^d^						
0	11,976 (88.5)	11,788 (91.7)	1,783 (91.1)	2,257 (91.6)	27,804 (90.3)	
1	1,073 (7.9)	718 (5.6)	127 (6.5)	142 (5.8)	2,060 (6.7)	
2	314 (2.3)	252 (2.0)	38 (1.9)	46 (1.9)	650 (2.1)	
3	162 (1.2)	95 (0.7)	10 (0.5)	18 (0.7)	285 (0.9)	<0.0001
**ASA classification**^**e**^						
1	2,680 (19.8)	3,383 (26.3)	528 (27.0)	519 (21.1)	7,110 (23.1)	
2	8,694 (64.3)	7,919 (61.6)	1,191 (60.8)	1,588 (64.5)	19,392 (63.0)	
3	2,151 (15.9)	1,551 (12.1)	239 (12.2)	356 (14.5)	4,297 (14.0)	<0.0001
**Methotrexate**	488 (3.6)	348 (2.7)	50 (2.6)	61 (2.5)	947 (3.1)	<0.0001

^a^ Abstention = 0 g/week. Low-to-moderate consumption = >0–168 g/week. High consumption = >168–252 g/week. Excessive consumption = >252 g/week.

^b^ Pearson’s chi-squared test

^c^ Annual income is presented in 1000 DKK (1000 DKK corresponds to approx. € 134 Euros)

^d^ Charlson Comorbidity Index

^e^ American Society of Anesthesiologists physical status classification

Abstainers were typically older, female and non-smoking and had more comorbidities than alcohol consumers, as indicated by higher scores on the Charlson Comorbidity Index and the American Society of Anesthesiologists physical status classification (Table 1). Abstainers were also most often obese. Low-to-moderate consumers were typically younger, non-smokers, overweight, and had fewer comorbidities than abstainers. Within the group of excessive alcohol consumption, 40.1% were not asked about their smoking status. A graphic overview of the alcohol consumption distribution is presented in a histogram in S1 Fig.

### Postoperative mortality

During the first 90 days, a total of 285 (0.9%) deaths were identified. The number increased to 694 (2.3%) after one year (Table 2). Abstainers had the highest absolute and relative numbers of deaths at both time points, while low-to-moderate consumers had the lowest relative number of all consumption groups. A high relative number of deaths was also seen among excessive consumers. However, a low absolute numbers of deaths were detected among high and excessive consumers due to their smaller group sizes.

**Table 2:** Number (%) of cases in the primary outcomes among 30,799 patients undergoing primary hip or knee arthroplasty, presented according to alcohol consumption levels and in total.

	Abstention^a^	Low-to-moderate^a^	High^a^	Excessive^a^	Total	P value^b^
**Mortality, 90 days**	170 (1.3)	69 (0.5)	16 (0.8)	30 (1.2)	285 (0.9)	<0.001
**Mortality, 1 year**	407 (3.0)	191 (1.5)	36 (1.8)	60 (2.4)	694 (2.3)	<0.001

^a^ Abstention = 0 g/week, low-to-moderate consumption = >0–168 g/week, high consumption = >168–252 g/week, excessive consumption = >252 g/week

^b^ Pearson’s chi-squared test

**Fig 1:**
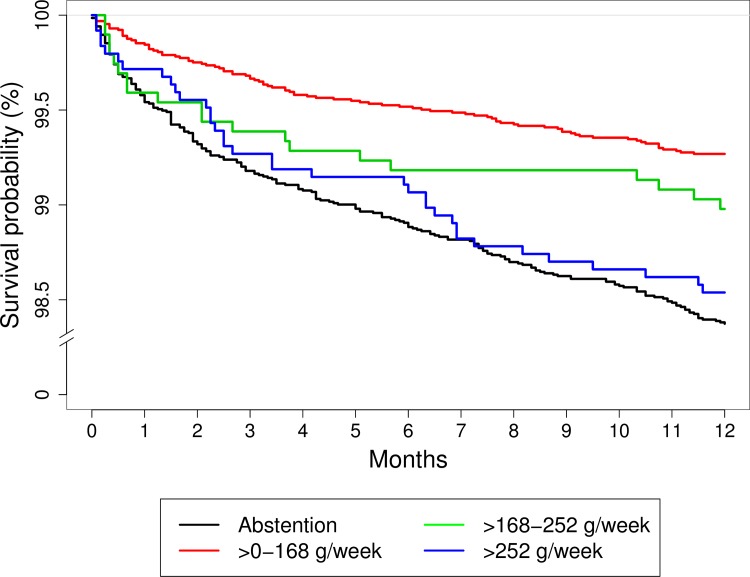
1-year survival rates after primary hip or knee arthroplasty plotted by the Kaplan Meier survival function. The survival rates of 30,799 patients, according to different levels of preoperative alcohol consumption during the first postoperative year after primary hip or knee arthroplasty in Denmark.

The Kaplan-Meier survival function showed that low-to-moderate consumers had better survival rates during the first year after surgery (Fig 1). The largest difference between low-to-moderate consumers and the other groups was seen during the first 30 days after surgery, as the survival rates in all other groups decreased notably. As seen in Table 2, abstainers had the poorest survival 1 year after surgery.

**Fig 2:**
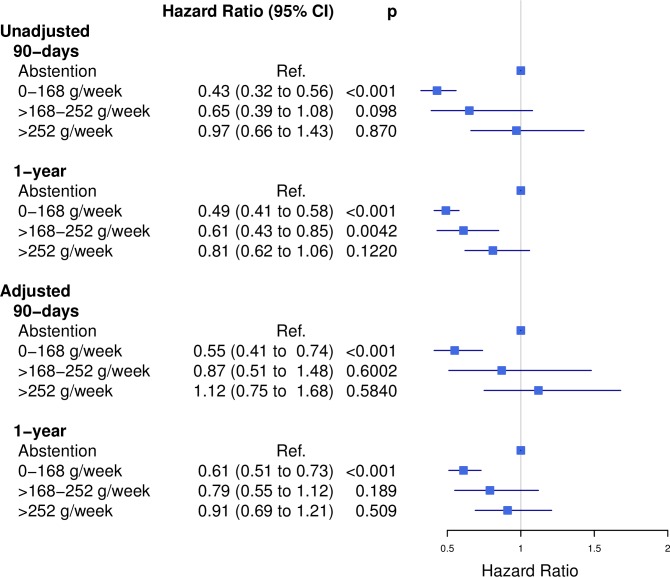
Postoperative mortality at 90 days and 1 year. Unadjusted and adjusted 90-day and 1-year risks of mortality among 30,799 arthroplasty patients with different preoperative levels of alcohol consumption.

Unadjusted models detected significantly lower risks of mortality after both 90 days (HR 0.43, 95% CI 0.32 to 0.56) and 1 year (HR 0.49, 95% CI 0.41 to 0.58) among low-to-moderate consumers when compared to abstainers (Fig 2). After adjusting for confounders, attenuation of the association between these groups was noted; yet, significantly lower risks for both 90-day (HR 0.55, 95% CI 0.41 to 0.74) and 1-year mortality (HR 0.61, 95% CI 0.51 to 0.73) were observed among low-to-moderate consumers. No other risk estimate differed significantly from abstainers in any adjusted model. Results of the supplementary analysis of 1-year mortality stratified by age-quartiles did not reveal significant differing risk estimates across consumption groups (S1 Table).

### Postoperative morbidity

In total, we identified 209 cases of cardiovascular disease and 270 cases of deep venous thrombosis during the first 30 days after surgery. A total of 514 patients acquired prosthetic infection during the 1-year follow-up (Table 3). A slightly higher cumulative incidence proportion of cardiovascular diseases 30 days after surgery was observed among abstainers (0.8%, [95% CI, 0.7% to 1.0%]), while deep venous thrombosis was most frequent among excessive consumers (1.3%, [95% CI, 0.8% to 1.7%]). High consumers had the highest cumulative incidence proportion of prosthetic infection after 1 year (2.6%, [95% CI, 1.9% to 3.3%]).

**Table 3:** Number of cases (n) and cumulative incidence ((%), [95% Confidence Intervals (CI)]) in the secondary outcomes among 30,799 patients undergoing primary hip or knee arthroplasty, presented according to alcohol consumption levels and in total.

	Abstention^a^	Low-to-moderate^a^	High^a^	Excessive^a^	Total
**Cardiovascular disease, 30 days.** Number of cases, Cumulative incidence ((%), [95% CI])	114, 0.8 [0.7 to 1.0]	69, 0.5 [0.4 to 0.7]	14, 0.7 [0.3 to 1.1]	12, 0.5 [0.2 to 0.8]	209, 0.7 [0.6 to 0.8]
**Deep venous thrombosis, 30 days.** Number of cases, Cumulative incidence ((%), [95% CI])	126, 0.9 [0.8 to 1.1]	100, 0.8 [0.6 to 0.9]	13, 0.7 [0.3 to 1.0]	31, 1.3 [0.8 to 1.7]	270, 0.9 [0.8 to 1.0]
**Prosthetic infection, 1 year.** Number of cases, Cumulative incidence ((%), [95% CI])	229, 1.7 [1.5 to 1.9]	184, 1.4 [1.2 to 1.6]	51, 2.6 [1.9 to 3.3]	50, 2.0 [1.5 to 2.6]	514, 1.7 [1.5 to 1.8]

^a^ Abstention = 0 g/week, low-to-moderate consumption = >0–168 g/week, high consumption = >168–252 g/week, excessive consumption = >252 g/week

**Fig 3:**
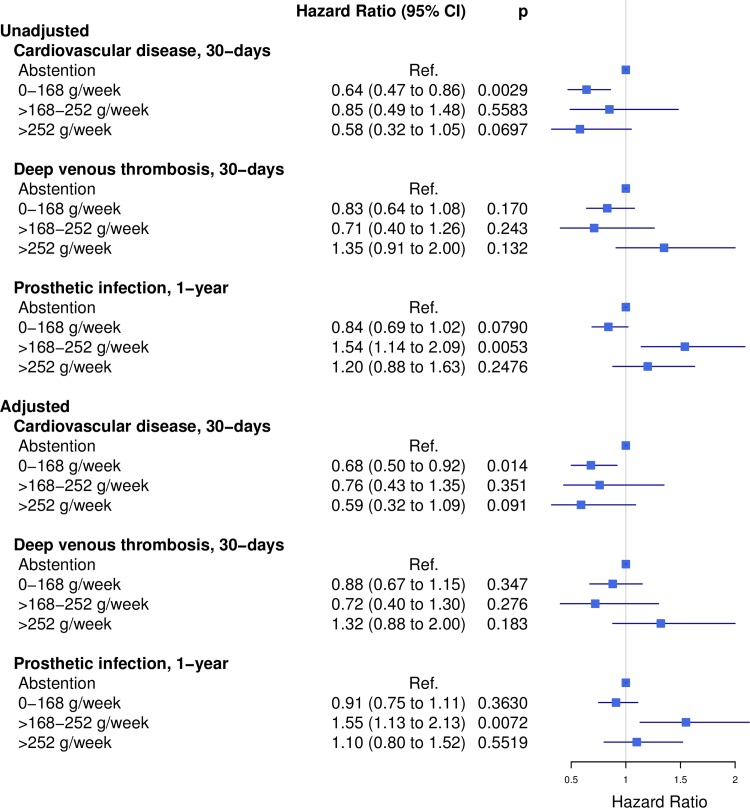
Risk of postoperative morbidity. Unadjusted and adjusted risks of prosthetic infection after 1 year and cardiovascular disease and deep venous thrombosis after 30 days among 30,799 arthroplasty patients with different preoperative levels of alcohol consumption.

The unadjusted risk estimates observed in all alcohol consumption groups differed generally more from abstainers than the adjusted risk estimates (Fig 3). The adjusted risk of cardiovascular disease after 30 days was significantly lower among patients with low-to-moderate consumption than among abstainers (HR 0.68, 95% CI 0.50 to 0.92). Other endpoints did not reveal any significant risk differences between these groups. The adjusted estimates showed an increased risk of prosthetic infection for high consumers compared to abstainers (HR 1.55, 95% CI 1.13 to 2.13).

### Sensitivity analysis

#### Postoperative mortality

Compared to the adjusted results, no notable differences were identified in any model when patients not asked about their smoking status were grouped as non-smokers (Sensitivity analysis 1, S2 Fig) or when abstainers not asked about smoking status were grouped as non-smokers and the remaining patients not asked about their smoking status were grouped as smokers (Sensitivity analysis 2, S2 Fig). A slight increase in the risk attributed to excessive consumers in 1-year mortality was observed in Sensitivity analysis 1, but no significant risk difference was seen when compared to abstainers.

#### Postoperative morbidity

Similarly, the risk estimates seen in the sensitivity analyses regarding morbidity did not differ notably from the originally adjusted results; consequently, no change in the direction of any result was detected (S3 Fig).

## Discussion

The main findings of this study were that arthroplasty patients with low-to-moderate preoperative alcohol consumption had lower risks of both 90-day and 1-year mortality when compared to patients who abstained from alcohol prior to surgery. Additionally, low-to-moderate consumers were associated with a lower risk of cardiovascular disease after 30 days compared to abstainers. These findings were observed in both unadjusted and adjusted models. There were no other differences in other endpoints between abstainers and low-to-moderate consumers, but when comparing abstainers to high consumers, a higher risk of prosthetic infection within the first year after surgery was observed among high consumers.

Our results regarding postoperative mortality corresponded to our hypothesis, as we observed a significantly decreased risk of postoperative mortality among low-to-moderate consumers compared to abstainers. However, this decreased risk was not completely recapitulated for postoperative morbidity. We expected no between-group difference, which was the case for deep venous thrombosis and prosthetic infection, but low-to-moderate consumers had a significantly lower risk of cardiovascular complications within 30 days after surgery. Our findings may suggest that low-to-moderate preoperative alcohol consumption reduces the risk of postoperative mortality, but the nature of the study makes it impossible to imply causality. This result may be driven by other aspects of health that are also associated with low-to-moderate alcohol consumption.

A range of postoperative complications has been studied in relation to different levels of alcohol consumption, but few studies have focused on the risk of these complications in relation to low-to-moderate preoperative consumption compared to complete abstention [3]. Decreased risks of several postoperative complications among low and moderate alcohol consumers when compared to abstainers has been reported, but there were no significant between-group differences [3]. Similarly, we observed decreased risks of all postoperative complications for low-to-moderate consumers when compared to abstainers, but only in the case of cardiovascular disease did the estimates differ significantly between these groups. Other studies have investigated the risk of adverse postoperative events among patients, yet with differing alcohol consumption thresholds. Among these, Jørgensen et al. compared consumption of more than 168 g/week with a lower consumption level and did not detect any risk differences between these groups in terms of general or alcohol-specific readmission rates after hip or knee arthroplasty [37]. However, Nath et al. detected increased risks of certain postoperative complications associated with consumption of more than approximately 200 g/week when compared to a lower consumption level but did not find a significantly increased risk of postoperative mortality [33]. The relationship between alcohol consumption and adverse postoperative events seems to differ depending on outcome, level of alcohol consumption, surgical populations, and several other potential factors. The general inconsistency of the findings observed in both our study and the literature suggests that this relationship may be highly complex.

Although the relationships between the specific levels of alcohol consumption and postoperative mortality and morbidity have been insufficiently studied, it may be suggested that low-to-moderate consumers have a lower risk of cardiovascular disease compared to abstainers, as low or moderate alcohol consumption may protect against adverse cardiovascular events [38]. This mechanism may also be a contributing factor for the observation of lower mortality risk among low-to-moderate consumers because cardiovascular diseases are reported to be a frequent cause of death after hip arthroplasty [39].

We did find an increased risk of prosthetic infection among high consumers compared to abstainers, which can potentially be linked to several abnormalities in the immune responses of chronic alcohol users that make them more susceptible to infections [40–42]. However, it is noteworthy that we did not detect any differences between abstainers and excessive consumers in our study, as this group included all consumption above 252 g/week. In contrast, a previous review of the literature detected increased risks of postoperative morbidity, with odds ratios ranging from 3.1 to 26.6 among alcohol abusers consuming at least 60 grams of alcohol per day [42], which corresponds to 420 g/week. The lack of difference in risk between abstainers and excessive consumers may indicate that heavy alcohol abusers in this study had consumption close to the lower limit. Most likely, true heavy consumers might not be candidates for surgery.

### Strengths and limitations

Large-scale observational study may often be the most suitable approach to detect late adverse outcomes in clinical practice [43]. The Danish Civil Personal Register number provides a unique opportunity to link data from several national registries, which enabled us to combine information on a large population at an individual level and ensured minimal loss to follow-up. Additionally, the use of individual pre- and postoperative health records that cover an extensive amount of time strengthens the results.

Another strength is the use of data on preoperative alcohol consumption collected by hospital staff shortly before surgery. Patients would generally be informed about possible interactions between alcohol and anesthetic drugs and may therefore be inclined to provide more accurate information on alcohol consumption than is given in general population studies. Furthermore, comprehensively adjusted risk estimates added strength to our study. Both the Charlson Comorbidity Index and the American Society of Anesthesiologists physical status classification were included to account for preoperative comorbidity status in the adjusted models. The Charlson Comorbidity Index addresses the individual preoperative history of comorbidities, and the American Society of Anesthesiologists physical status classification describes the physical status of the patient immediately prior to surgery. The American Society of Anesthesiologists physical status classification and the Charlson Comorbidity Index may predict different types of postoperative complications in patients undergoing spinal surgery [44], which supports the inclusion of both measures.

Some limitations are related to this study. First, the consumption groups differed significantly in all baseline characteristics, which may allow residual confounding in spite of confounder adjustment. Other unobserved confounding might also remain unadjusted. Second, as patients with excessive alcohol consumption are generally recommended to comply with a preoperative period of abstinence, some patients designated as abstainers in our study may actually be former excessive consumers. Data describing any former history of alcohol consumption could not be obtained. Consequently, we were not able to determine this specific proportion of patients or its implications. Preoperative abstinence may also be a result of poor state of health or medication use, as proposed by the sick-quitter hypothesis [19]. Using abstainers as reference might therefore lead to abstainer bias where the protective association seen in low risk drinkers are overestimated when compared to abstainers [45]. This hypothesis might apply to some abstainers in our study, as this group showed larger proportions of American Society of Anesthesiologists physical status classification of 3, Charlson Index of 3, and methotrexate use at baseline. However, as recommended by Rehm et al. [45], this possibility was presumably addressed through confounder adjustment.

Third, although the use of administrative databases reduces the risk of missing patients and drop-out during follow-up, risks of inadequate registration, misclassification, and insufficient encoding still exist [4,46]. When compared to other sources of data, certain diagnoses appear to be underreported in the Danish National Patient Register [5,47]. Furthermore, diagnoses are only registered in the Danish National Patient Register when patients are hospitalized. Underreporting of diagnoses may result in insufficient comorbidity adjustment and underestimated risks of postoperative morbidity. Furthermore, underreporting is a common problem with self-reported alcohol consumption due to impression management or recall bias [48,49]. Underreported consumption can result in misclassification and is most likely to affect younger and low-risk drinkers [50]. Some underestimation might therefore be associated to low-to-moderate consumers in our study. However, as our study population is 45 years or older and as data on alcohol consumption are collected prior to undergoing surgery, underreporting may be of less significance than when studying younger populations. Finally, 40.1% of the patients who reported excessive alcohol consumption were not asked about smoking status. There are reasons to believe that the true number of smokers within this group may be higher than reported because alcohol consumers are often smokers [27]. Patients not asked about their smoking status were therefore categorized as smokers and sensitivity analyses were conducted to test this assumption. These analyses did not reveal any significant changes in any risk estimate when this group was handled as non-smokers or when only abstainers were handled as non-smokers. This indicates that our main results were robust.

### Implications

This study contributes to the limited knowledge on the relationship between low-to-moderate alcohol consumption or complete abstention and postoperative mortality and morbidity in an orthopedic population. To identify the best approach for addressing alcohol-related risks in surgical populations, it is important to know whether preoperative abstinence is better than modest consumption [51]. Our study may indicate that low-to-moderate consumers have better survival rates at 90 days and 1 year after primary hip or knee arthroplasty than complete abstainers. Moreover, a lower risk of cardiovascular disease after 30 days was associated with low-to-moderate consumption. Our results indicate that a consumption level greater than 168 g/week may be associated with increased risks of adverse outcomes, which supports the preoperative guidance physicians provide to patients on alcohol consumption prior to primary hip or knee arthroplasty. However, current preoperative alcohol recommendations address mainly patients who are excessive consumers, which suggests a preoperative period of abstention [1]. Our findings could be of relevance in a discussion on whether these recommendations should be revised, taking into account that a low level of alcohol consumption prior to hip or knee arthroplasty may not be associated to an increased risk of postoperative mortality, cardiovascular disease, deep venous thrombosis or prosthetic infection. Nevertheless, randomized trials are needed to confirm our results and to increase our understanding of causal mechanisms. Furthermore, additional research is needed in conjunction with data on former alcohol consumption so that the patients who may benefit from such recommendations could be identified more precisely. Finally, future research should investigate whether our findings are applicable to other surgical populations.

## Supporting information

S1 Table Hazard ratios [and 95% Confidence Intervals] for 1-year mortality among 30,799 patients undergoing primary hip or knee arthroplasty, according to alcohol consumption levels, stratified by age-quartiles groups. (DOCX)

S1 Fig Distribution of alcohol consumption in the study population. The distribution of alcohol consumption of 30,799 arthroplasty patients presented in categories of weekly consumption in grams (g/week).
(PDF)

S2 Fig Sensitivity analyses–Postoperative mortality. Sensitivity analyses of mortality risks among 30,799 arthroplasty patients with different preoperative levels of alcohol consumption, where patients not asked about their smoking status were grouped as non-smokers (Sensitivity analysis 1) and abstaining patients not asked about their smoking status were grouped as non-smokers, while the remaining patients not asked were grouped as smokers (Sensitivity analysis 2).
(PDF)

S3 Fig Sensitivity analyses–Postoperative morbidity. Sensitivity analyses of morbidity risks among 30,799 arthroplasty patients with different preoperative levels of alcohol consumption, where patients not asked about their smoking status were grouped as non-smokers (Sensitivity analysis 1) and abstaining patients not asked about their smoking status were grouped as non-smokers, while the remaining patients not asked were grouped as smokers (Sensitivity analysis 2).
(PDF)
