# Supplementary material for: Alcohol consumption and the risk of postoperative mortality and morbidity after primary hip or knee arthroplasty – A register-based cohort study
Source: PLoS One. 2017 Mar 17;12(3):e0173083. doi: 10.1371/journal.pone.0173083 (PMC5357001; doi:10.1371/journal.pone.0173083)
Supplement: S1 Table — (DOCX) [file pone.0173083.s001.docx]

| **S1 Table** Hazard ratios [and 95% Confidence Intervals] for 1-year mortality^a^ among 30,799 patients undergoing primary hip or knee arthroplasty, according to alcohol consumption levels, stratified by age-quartiles groups. | | | | | |
| --- | --- | --- | --- | --- | --- |
|  | **45 to 62.4 years** | **62.5 to 69.2 years** | **69.3 to 76.2 years** | **Over 76.2 years** |  |
| **Abstention**^b^ | 1 | 1 | 1 | 1 |  |
| **Low-to-moderate**^b^ | 0.48 [0.26 to 0,88] | 0.80 [0.48 to 1.35] | 0.79 [0.53 to 1.18] | 0.56 [0.44 to 0.71] |  |
| **High**^b^ | 0.28 [0.07 to 1.19] | 1.55 [0.78 to 3.08] | 0.98 [0.47 to 2.02] | 0.65 [0.37 to 1.16] |  |
| **Excessive**^b^ | 0.52 [0.23 to 1.14] | 1.11 [0.55 to 2.25] | 1.23 [0.63 to 2.41] | 0.91 [0.61 to 1.35] |  |
| ^a^ Estimated by cox regression models, adjusted by age, sex, smoking status, BMI, annual income, Charlson Comorbidity Index, American Society of Anesthesiologists physical status classification, operation type and preoperative use of methotrexate  ^b^ Abstention = 0 g/week, low-to-moderate consumption = >0-168 g/week, high consumption = >168-252 g/week, excessive consumption = >252 g/week | | | | | |
