# Supplementary figures and images for: Alcohol consumption and the risk of postoperative mortality and morbidity after primary hip or knee arthroplasty – A register-based cohort study
Source: PLoS One. 2017 Mar 17;12(3):e0173083. doi: 10.1371/journal.pone.0173083 (PMC5357001; doi:10.1371/journal.pone.0173083)

# Distribution of alcohol consumption in the study population

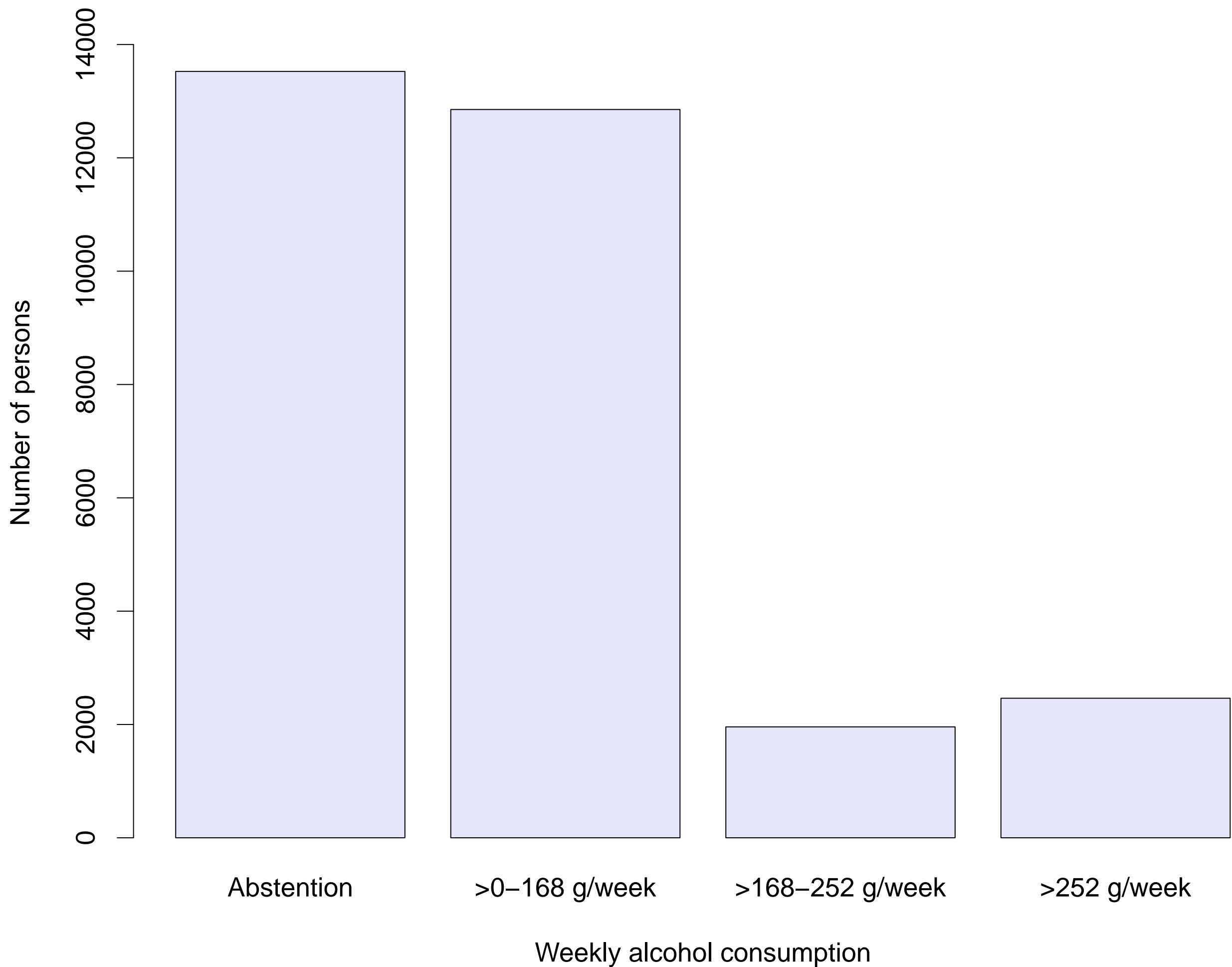

Supplement: S1 Fig — The distribution of alcohol consumption of 30,799 arthroplasty patients presented in categories of weekly consumption in grams (g/week). (PDF) [file pone.0173083.s002.pdf]

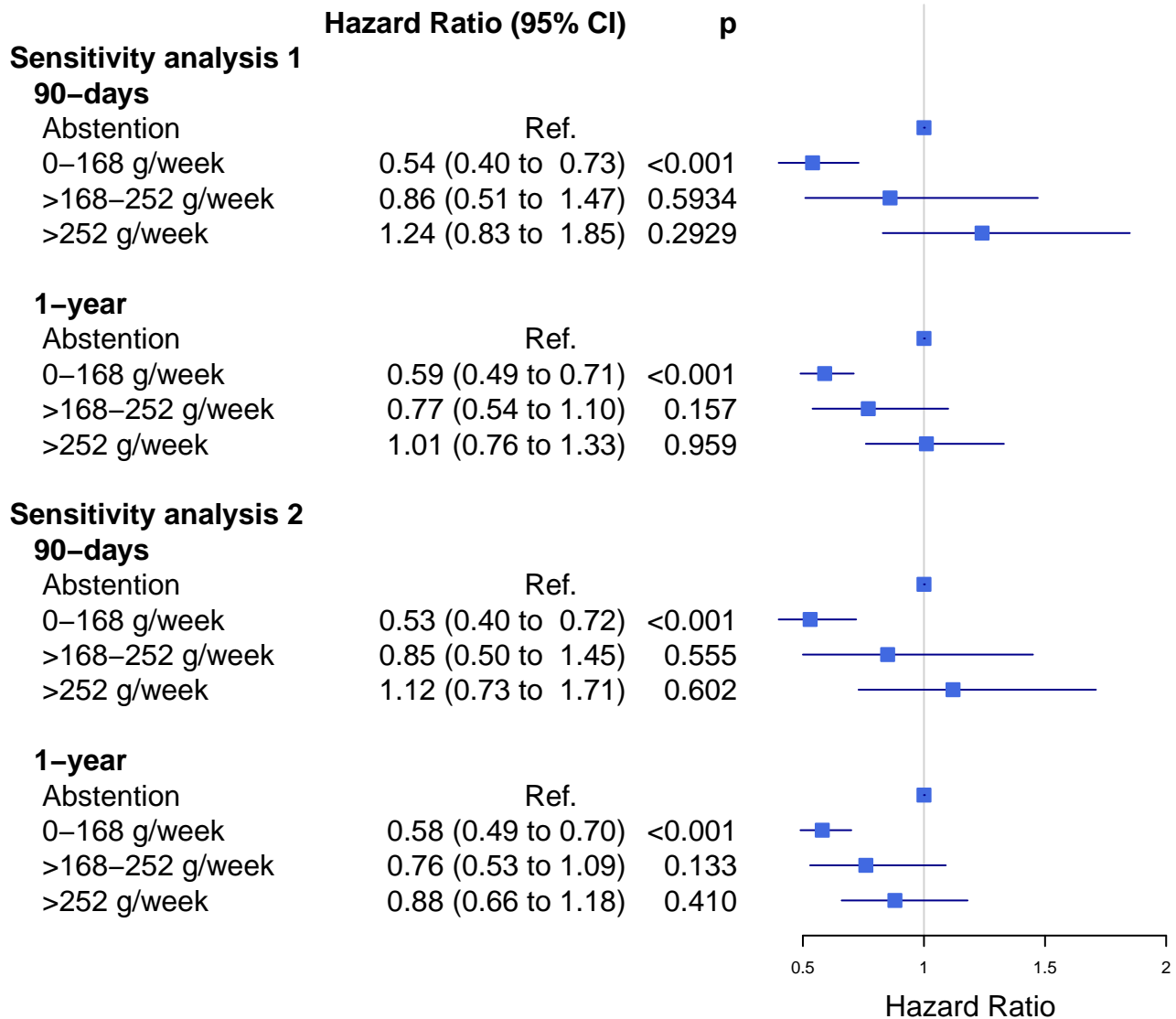

Supplement: S2 Fig — Sensitivity analyses of mortality risks among 30,799 arthroplasty patients with different preoperative levels of alcohol consumption, where patients not asked about their smoking status were grouped as non-smokers (Sensitivity analysis 1) and abstaining patients not asked about their smoking status were grouped as non-smokers, while the remaining patients not asked were grouped as smokers (Sensitivity analysis 2). (PDF) [file pone.0173083.s003.pdf]
