# Supplementary material for: Alcohol consumption and the risk of postoperative mortality and morbidity after primary hip or knee arthroplasty – A register-based cohort study
Source: PLoS One. 2017 Mar 17;12(3):e0173083. doi: 10.1371/journal.pone.0173083 (PMC5357001; doi:10.1371/journal.pone.0173083)

	Hazard Ratio (95% CI)	p
**Sensitivity analysis 1**		
**Cardiovascular disease, 30−days**		
Abstention	Ref.	
0−168 g/week	0.66 (0.48 to 0.90)	0.0094
>168−252 g/week	0.74 (0.41 to 1.31)	0.2965
>252 g/week	0.61 (0.33 to 1.11)	0.1051
**Deep venous thrombosis, 30−days**		
Abstention	Ref.	
0−168 g/week	0.88 (0.67 to 1.16)	0.369
>168−252 g/week	0.73 (0.40 to 1.31)	0.286
>252 g/week	1.30 (0.87 to 1.95)	0.200
**Prosthetic infection, 1−year**		
Abstention	Ref.	
0−168 g/week	0.90 (0.74 to 1.10)	0.3088
>168−252 g/week	1.54 (1.11 to 2.12)	0.0088
>252 g/week	1.15 (0.84 to 1.57)	0.3761
**Sensitivity analysis 2**		
**Cardiovascular disease, 30−days**		
Abstention	Ref.	
0−168 g/week	0.65 (0.48 to 0.90)	0.0083
>168−252 g/week	0.73 (0.41 to 1.30)	0.2884
>252 g/week	0.55 (0.30 to 1.02)	0.0596
**Deep venous thrombosis, 30−days**		
Abstention	Ref.	
0−168 g/week	0.90 (0.69 to 1.19)	0.468
>168−252 g/week	0.75 (0.42 to 1.36)	0.345
>252 g/week	1.44 (0.94 to 2.21)	0.096
**Prosthetic infection, 1−year**		
Abstention	Ref.	
0−168 g/week	0.89 (0.73 to 1.10)	0.281
>168−252 g/week	1.52 (1.10 to 2.11)	0.010
>252 g/week	1.09 (0.78 to 1.51)	0.613

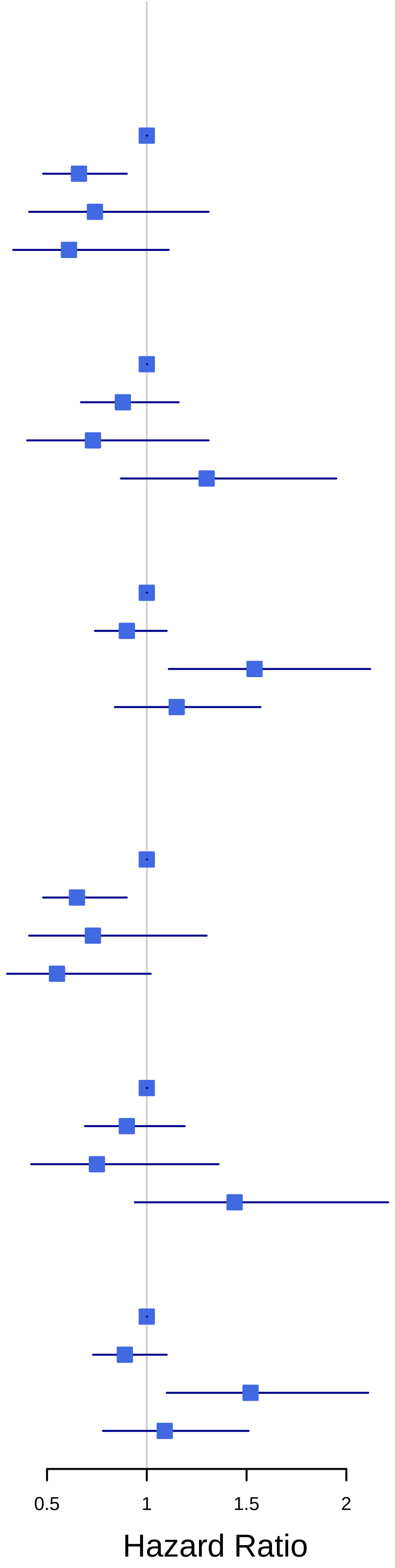

Supplement: S3 Fig — Sensitivity analyses of morbidity risks among 30,799 arthroplasty patients with different preoperative levels of alcohol consumption, where patients not asked about their smoking status were grouped as non-smokers (Sensitivity analysis 1) and abstaining patients not asked about their smoking status were grouped as non-smokers, while the remaining patients not asked were grouped as smokers (Sensitivity analysis 2). (PDF) [file pone.0173083.s004.pdf]
